# VEGF Receptor-2 (Flk-1) Overexpression in Mice Counteracts Focal Epileptic Seizures

**DOI:** 10.1371/journal.pone.0040535

**Published:** 2012-07-12

**Authors:** Litsa Nikitidou, Irene Kanter-Schlifke, Joke Dhondt, Peter Carmeliet, Diether Lambrechts, Mérab Kokaia

**Affiliations:** 1 Experimental Epilepsy Group, Wallenberg Neuroscience Center, BMC A-11, Lund University Hospital, Lund, Sweden; 2 Laboratory of Angiogenesis and Neurovascular link, Vesalius Research Center, VIB, Leuven, Belgium; 3 Laboratory of Angiogenesis and Neurovascular link, Vesalius Research Center, KU Leuven, Leuven, Belgium; University of Jaén, Spain

## Abstract

Vascular endothelial growth factor (VEGF) was first described as an angiogenic agent, but has recently also been shown to exert various neurotrophic and neuroprotective effects in the nervous system. These effects of VEGF are mainly mediated by its receptor, VEGFR-2, which is also referred to as the fetal liver kinase receptor 1 (Flk-1). VEGF is up-regulated in neurons and glial cells after epileptic seizures and counteracts seizure-induced neurodegeneration. *In vitro*, VEGF administration suppresses ictal and interictal epileptiform activity caused by AP4 and 0 Mg^2+^ via Flk-1 receptor. We therefore explored whether increased VEGF signaling through Flk-1 overexpression may regulate epileptogenesis and ictogenesis *in vivo*. To this extent, we used transgenic mice overexpressing Flk-1 postnatally in neurons. Intriguingly, Flk-1 overexpressing mice were characterized by an elevated threshold for seizure induction and a decreased duration of focal afterdischarges, indicating anti-ictal action. On the other hand, the kindling progression in these mice was similar to wild-type controls. No significant effects on blood vessels or glia cells, as assessed by Glut1 and GFAP immunohistochemistry, were detected. These results suggest that increased VEGF signaling via overexpression of Flk-1 receptors may directly affect seizure activity even without altering angiogenesis. Thus, Flk-1 could be considered as a novel target for developing future gene therapy strategies against ictal epileptic activity.

## Introduction

Vascular endothelial growth factor (VEGF or VEGF-A) is a member of homodimeric glycoproteins and was initially shown to increase vascular permeability in tumor ascites fluid [1]. Since its discovery, VEGF has been found to have various roles in normal and pathologic conditions in the brain. For example, VEGF enhances neuronal proliferation [2], [3], [4], [5], [6], [7], survival [8], [9], [10], [11], [12] and axonal outgrowth [13], [14]. Both VEGF-A and VEGF-B, as well as their receptors, VEGFR-1, VEGFR-2 and neuropilin, are widely expressed in the brain with differential expression in distinct population of cells [15]. Immunohistological evaluation reveals their co-localization in all types of neural cells, including pyramidal neurons of the cortex and hippocampus, both in rodents after status epilepticus (SE), and human tissue resected from patients with focal cortical dysplasia-induced intractable epilepsy [15], [16], [17]. This suggests possible autocrine/paracrine mechanisms of action, and possible role in epileptogenesis and/or ictogenesis.

In the hippocampus, VEGF protects hippocampal neurons after hypoxia [10], glutamate excitotoxicity [18] and SE [16], [17], [19]. Following electroconvulsive seizures, the levels of VEGF mRNA are increased in brain areas susceptible to cell loss, such as the hippocampus [20]. In addition, VEGF protein is up-regulated 24 h after pilocarpine-induced seizures [17]. *In vitro*, hippocampal slices from rats with recurrent spontaneous seizures show reduced bicuculline-induced epileptiform discharges after VEGF application [21]. Moreover, VEGF was shown to decrease both ictal and interictal activity induced by AP4 and 0 Mg^2+^ in rat hippocampal slices [22]. The neurotrophic and neuroprotective effects of VEGF are predominantly mediated by VEGF receptor-2, also called fetal liver kinase receptor 1 (Flk-1) [13], [23], [24], or kinase insert-domain containing receptor (KDR) in humans [25].

The mechanisms of action of VEGF through the Flk-1 receptor are still not completely understood. The pathway of signal transduction seems to be mediated by phosphatidylinositol 3′-kinase/Akt (PI3K/Akt), phospholipase C-gamma/protein kinase C (PLC-γ/PKC) and mitogen-activated protein kinase/extracellular signal-regulated protein kinase (MEK/ERK) pathways [26], [27], [28]. The neuroprotective effect is thought to be mediated by activation of the PI3K/Akt cascade, while the effects on axonal outgrowth and neuroproliferation are most likely dependent on the PKC- and ERK- dependent pathways.

The main objective of the present study was to investigate whether Flk-1 overexpression in transgenic mice, mimicking increased Flk-1 expression in the temporal lobe after epileptic seizures, would exert direct regulatory action on epileptogenesis and/or ictogenesis.

## Methods

### Ethics Statement

All experimental procedures were approved by the local Malmö/Lund Ethical Committee for Experimental Animals (Ethical permit number M87-06) and were performed according to the guidelines of the Swedish Animal Welfare Agency and in agreement with international guidelines.

### Animals

Five male transgenic mice expressing murine Flk-1 transgene in postnatal neurons under the Thy-1.2 promoter (Thy1-Flk-1 OE) and 10 male FvB controls were used [29]. Only male animals were used to exclude effects of fluctuating female hormones on kindling epileptogenesis [30], [31], [32]. The animals weighing 20 g at the beginning of the experiment, had *ad libitum* access to food and water, and were housed at a 12 hour light/dark cycle.

### Implantation of electrodes

Animals were anesthetized by intraperitoneal injection of ketamine (Ketalar, Pfizer; 80 mg/kg) and xylazine (Sigma-Aldrich, Stockholm, Sweden; 15 mg/kg). A bipolar stainless steel stimulation/recording electrode (Plastics One, Roanoke, VA) was stereotaxically (David Kopf Instruments, Tujunga, CA) implanted in the hippocampus at the following coordinates: AP -2.9, ML -3.0, DV -3.0 from bregma, midline and dura, respectively [33]. A reference electrode was placed in the temporal muscle. Proximal electrode sockets were inserted into a plastic pedestal (Plastics One, Roanoke, VA) and fixed on the skull with dental cement (Kemdent, Wiltshire, UK). The animals were allowed to recover for one week before electrical stimulation.

### Electrical kindling stimulation

On the first 3–5 days of kindling the individual threshold was measured by starting stimulations at 10 µA and increasing the current intensity in 10 µA steps (1 ms square wave pulse, 100 Hz) until a focal EEG epileptiform afterdischarge (AD) of at least 5 sec duration was observed. The threshold was determined once a day until ADs were evoked three times by the same minimal current intensity. Thereafter animals were stimulated at their individual threshold current once a day. Stimulation-induced behavioral seizures were scored according to the Racine scale [34]: Stage 0 – no behavioral changes; Stage 1 – facial twitches; Stage 2 – chewing and head nodding; Stage 3 – unilateral forelimb clonus; Stage 4 – rearing, body jerks, bilateral forelimb clonus; Stage 5 – imbalance. EEG was recorded on a MacLab system (ADInstruments, Bella Vista, Australia) 1 min before and 1 min after electrical stimulation. Animals were considered fully kindled when three stage 5 seizures were obtained.

### Immunohistochemistry

Forty-eight hours after last stimulation-induced stage 5 seizure, animals were deeply anesthetized with pentobarbital and perfused through the ascending aorta with 0.9% NaCl followed by ice-cold 4% paraformaldehyde. Brains were post-fixed overnight at 4°C, cryoprotected in 20% sucrose (overnight at 4°C) and 30 µm coronal sections were cut on a microtome. The sections were stored in a cryoprotective solution at −20°C until use. For validation of electrode location sections were stained with 0.5% cresyl violet (Sigma-Aldrich, Stockholm, Sweden).

For glial fibrillary acidic protein (GFAP) immunostaining, slices were first rinsed in 0.02 M KPBS followed by preincubation with 5% normal goat serum (NGS) in 0.25% TKPBS and incubation with primary antibody mouse anti-GFAP (Sigma-Aldrich, Stockholm, Sweden; 1∶500) overnight at room temperature (RT). The next day slices were rinsed with 0.02 M KPBS and incubated 2 hours with secondary antibody FITC-goat anti-mouse (Jackson Immunoresearch, Suffolk, UK; 1∶400). Slices were rinsed once more and mounted on coated slides and coverslipped. The number of GFAP positive cells in the hilus was quantified by counting in Flk-1 OE mice (n = 3) and control mice (n = 3). From each animal three sections from the dorsal hippocampus were quantified bilaterally. The CA4 region was not included in the counting. Counting was conducted on Olympus BX61 microscope, while pictures were obtained with a Leica TCS SP2 confocal microscope.

Three different immunohistochemical stainings were performed with brain slices that did not undergo kindling stimulations; Flk-1 (WT n = 2; Flk-1 OE n = 2); VEGF (WT n = 2; Flk-1 OE n = 2) and glucose transporter 1, Glut1 (WT n = 4; Flk-1 OE n = 4). The brains were removed from the skull, fixed in 2% PFA and embedded in tissue-freezing medium (Tissue-Tek®, Sakura, Alphen aan den Rijn, Netherlands). The 10 µm coronal sections were incubated at RT overnight with primary antibodies directed against Glut1 (Santa Cruz Biotechnology, Santa Cruz, CA; 1∶20), VEGF (Santa Cruz Biotechnology, Santa Cruz, CA; 1∶20) and Flk-1 (R&D Systems, Abingdon, UK; 1∶200). Co-labeling with neuronal nuclei, NeuN (Millipore, Brussels, Belgium; 1∶500) was carried out with VEGF and Flk-1 stainings. Subsequently, the sections were incubated with fluorescently-labeled secondary antibodies Alexa 488 or 568 (Molecular Probes, Eugene, Oregon; 1∶200) for 2 h or with biotin-labeled IgG followed by amplification with a signal amplification system (Streptavidin-HRP-Fluorescein) for VEGF and Flk-1. Blood vessel area and density in the hippocampus were assessed using a Zeiss Axioplan microscope with KS300 image analysis software. VEGF and Flk-1 staining was visualized using a Zeiss LSM510 confocal microscope.

### RNA extraction, cDNA preparation and real-time PCR

To extract RNA, hippocampi were dissected (WT n = 3; Flk-1 OE n = 4) and homogenized in RLT lysis buffer (Qiagen, Venlo, Netherlands) using a FastPrep24 system (MP Biomedicals, Illkirch, France). RNA was extracted with a DNase digestion (Qiagen, Venlo, Netherlands) and was transcribed to cDNA by the QuantiTect reverse transcription kit (Qiagen, Venlo, Netherlands) according to the manufacturer's instructions. Gene expression was assessed by the 7500 Fast Real-Time PCR system (Applied Biosystems, Halle, Belgium) and cDNA was normalized to β-actin expression levels, with the following TaqMan gene expression assays: β-actin Mm00607939_s1; Flk1 Mm01222419_m1; VEGF Mm00437304_m1.

### Statistical analysis

Statistical analysis of data was performed using Student's unpaired *t*-test or one-way ANOVA. Differences between the group data were considered significant at *p*<0.05. Data are presented as mean ±SEM. The investigator conducting the behavioral grading of animals, EEG analysis or histological and PCR analysis was unaware of the group identity of individual animals.

## Results

### Flk-1 OE increases threshold and shortens duration of focal seizures

**Figure 1:**
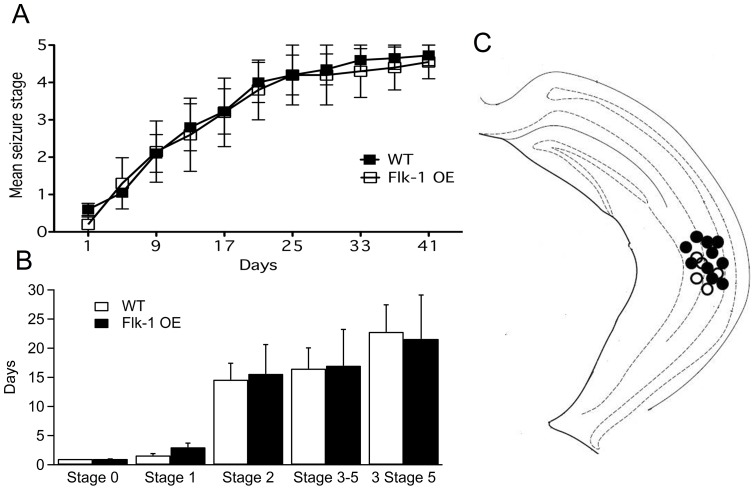
Epileptogenesis is not alternated by overexpression of Flk-1. (A) Progression of kindling: mean stage during each kindling stimulation analyzed with one-way ANOVA. (B) Number of stimulations needed to reach different behavioral seizure stages. (C) Placement of electrodes for electrical stimulation in the hippocampus was verified by cresyl violet staining at the end of the experiments. Solid black dots for WT mice and black rings for Flk-1 OE mice.

**Figure 2:**
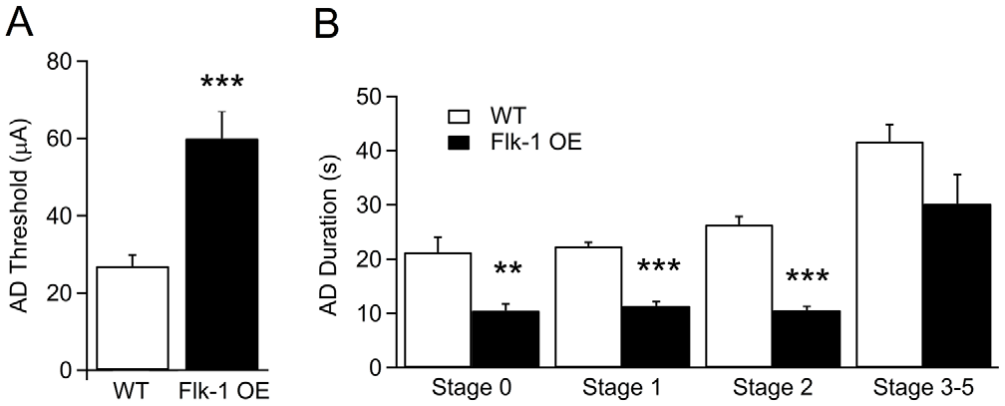
Flk-1 OE mice have higher threshold for seizure-induction and exhibit shorter afterdischarge durations of seizures. (A) Mean afterdischarge (AD) threshold during focal epileptiform activity of at least 5 sec duration measured one week after electrode implantation of WT and Flk-1 OE mice. (B) AD duration for different seizure stages during kindling stimulations. Values are presented as mean ±SEM, ** *p*<0.01 and *** *p*<0.001.

The location of the implanted stimulation/recording electrodes was confirmed in hippocampal sections with standard cresyl violet staining. All electrode tips were placed within the expected same area of the hippocampus in the two groups (Fig. 1C). Seizures arising during initial kindling stimulations were focal without any behavioral manifestations. Subsequent stimulations induced gradual development of generalized seizures with increasing severity of behavioral manifestations (Fig. 1A, one-way ANOVA and 1B). There was no significant difference between the Flk-1 OE and WT mice in the number of stimulations needed to reach each stage of kindling, including the fully kindled stage (three stage 5 seizures) that was reached after 21.6±7.5 and 22.8±4.6 days, respectively (Fig. 1B). Thus, overexpression of Flk-1 did not alter epileptogenesis *per se*. However, the threshold for seizure induction was more than twice as high in Flk-1 OE mice as compared to WT animals (WT 27.0±2.9 µA; Flk-1 OE 60.0±7.1 µA; *p*<0.001) (Fig. 2A). Also, the AD duration of focal stages was greatly shorter (less than half) in Flk-1 OE animals (Stage 0: WT 21.3±2.8 sec, Flk-1 OE 10.5±1.2 sec; Stage 1: WT 22.4±0.7 sec, Flk-1 OE 11.4±0.8 sec; Stage 2: WT 26.4±1.5 sec, Flk-1 OE 10.6±0.7 sec; *p*<0.01), but remained unaltered between the groups in more generalized stages (Stage 3–5: WT 41.7±3.1 sec, Flk-1 OE 30.2±5.4 sec; *p*>0.05) (Fig. 2B). Taken together, these data suggest that Flk-1 OE strongly suppresses focal seizure activity.

### Flk-1 OE mice display elevated levels of Flk-1 and VEGF

**Figure 3:**
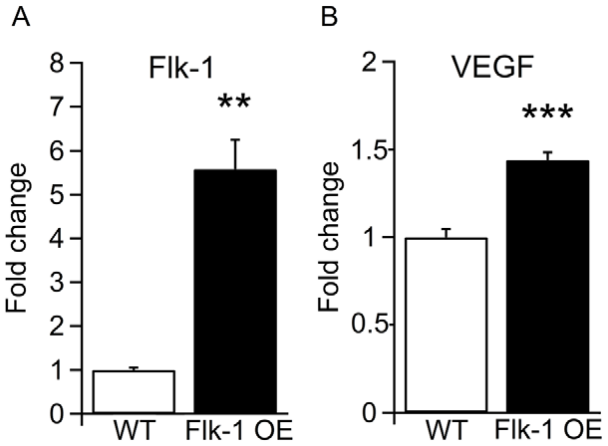
Real-time-PCR shows high levels of Flk-1 and VEGF in the hippocampus of Flk-1 OE mice. (A) Flk-1 expression was 5.58 fold higher in Flk-1 OE mice. (B) VEGF levels in the hippocampus of animals with Flk-1 OE were 1.44 fold higher than WT mice. The cDNA was normalized to β-actin expression levels. Values are presented as mean ± SEM, ** *p*<0.01 and *** *p*<0.001.

**Figure 4:**
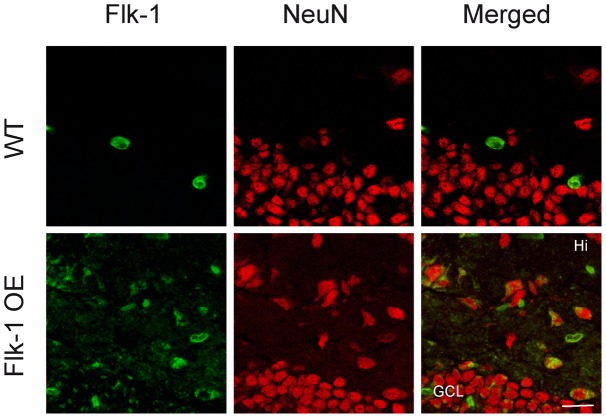
Immunoreactivity for Flk-1 in the hippocampal slices. Immunoreactivity for VEGF, NeuN and merged in the granule cell layer (GCL) and hilus (Hi) of the dentate gyrus. Flk-1 is barely detected in the hippocampus of wild-type (WT) mice, however the few cells that express Flk-1 are co-labeled with neuronal nuclei (NeuN) protein. In Flk-1 OE mice the Flk-1 immunoreactivity is mostly detected on the plasma membrane of the cells (indicated by the arrowheads). Scale bar is set to 20 µm.

The overexpression of Flk-1 in the transgenic mice was confirmed by real-time-PCR (Fig. 3A) and immunohistochemistry (Fig. 4). With immunostainings we just merely demonstrate expression pattern of the proteins, while using a more quantitative approach with real-time-PCR to estimate possible changes. The quantification of hippocampal mRNA showed a 5.58 fold increase (*p*<0.01) of Flk-1 in Flk-1 OE mice compared to WT (Fig. 3A). These quantitative results of real-time-PCR were supported by non-quantitative analysis of immunoreactivity, whereby the Flk-1 immunoreactivity was detected in numerous hippocampal neurons of Flk-1 OE mice, while WT mice displayed very few cells expressing Flk-1 (Fig. 4).

**Figure 5:**
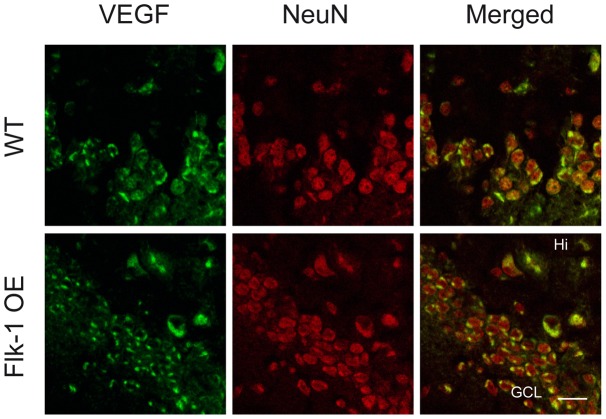
Vascular endothelial growth factor (VEGF) is expressed by neurons in the hippocampus. Figure shows immunoreactivity of VEGF, NeuN and merged in the granule cell layer (GCL) and hilus (Hi) of the dentate gyrus. In both controls and Flk-1 OE mice, neurons in the hippocampus express VEGF. Scale bar is set to 20 µm.

We hypothesized that Flk-1 overexpression could have led to compensatory changes and thereby decreased expression of its ligand, the VEGF. Therefore, we performed real-time-PCR (Fig. 3B) and immunohistochemistry (Fig. 5) to assess VEGF expression levels. Unexpectedly, VEGF mRNA expression was 1.44 fold higher in the hippocampus of Flk-1 OE mice compared to control mice (*p*<0.001) (Fig. 3B). Non-quantitative analysis of VEGF immunoreactivity showed VEGF positive neurons both in Flk-1 OE mice and in WT mice (Fig. 5). Taken together, our data suggest increased VEGF expression in the hippocampus of Flk-1 OE mice.

### Unaltered blood vessel densities in Flk-1 OE mice in the hippocampus

**Figure 6:**
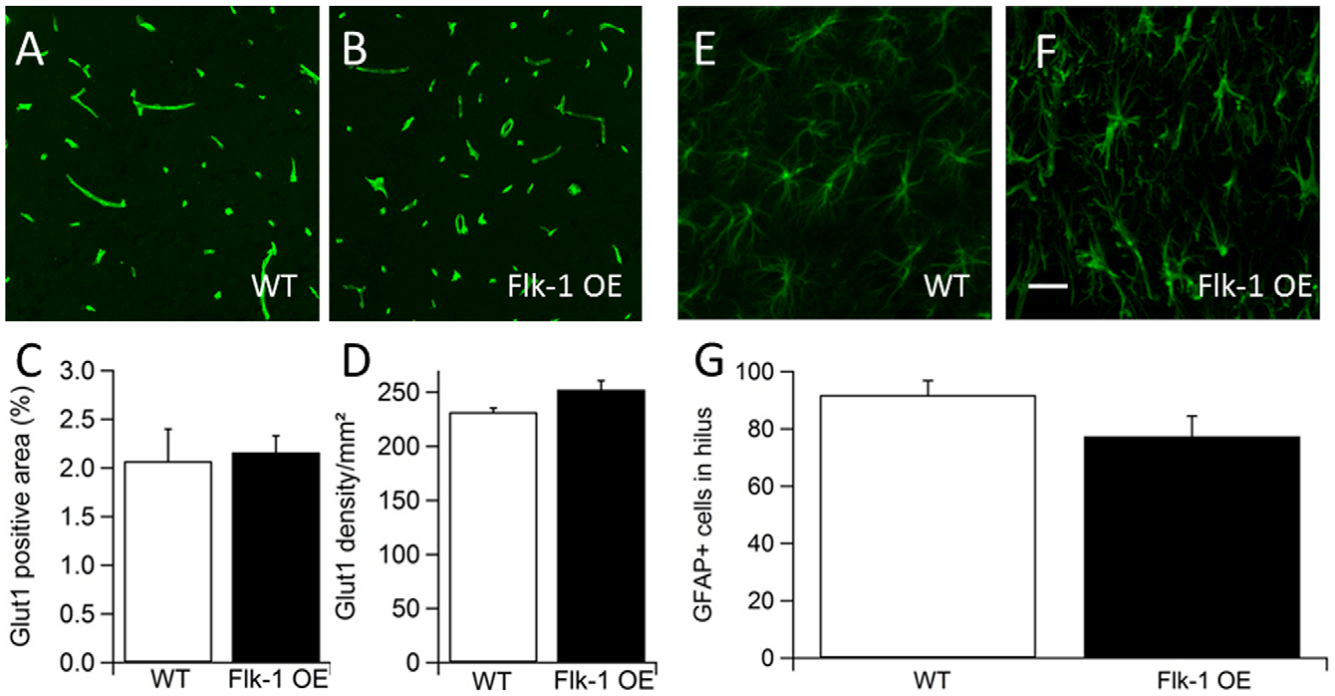
Glucose transporter 1 (Glut1) immunohistochemistry and quantification of blood vessels in the hippocampal slices. (A) Intensity and distribution of Glut-1 immunostaining is similar in the hippocampus of Flk-1 OE mice compared to (B) controls; Quantification of blood vessel area (C), as well as the blood vessel density (D) showed no significant differences (*p*>0.05) between the groups. The number of glial fibrillary acidic protein (GFAP)-positive cells in wild-type mice (E) and Flk-1 OE mice (F) in the hilus of the dentate gyrus after kindling are unaltered (G). Values are presented as mean ±SEM. Scale bar is set to 30 µm.

It is well known that VEGF can increase the vasculogenesis and angiogenesis [35], [36], [37] through activation of Flk-1. Since both VEGF and Flk-1 were found to be up-regulated in Flk-1 OE mice, we asked whether Flk-1 OE mice exhibit altered quantity and/or morphology of blood vessels. We quantified the area and density of blood vessels positive for glucose transporter 1 (Glut1) in the hippocampus (Fig. 6A–D). The Glut1 immunohistochemistry was performed in animals that did not undergo kindling stimulation, since it is known that that the expression of Glut1 is regulated by epileptic seizures [38]. In both groups about 2% of the hippocampus were Glut1 positive (WT 2.1±0.3%; Flk-1 OE 2.2±0.2%) (Fig. 6C), and blood vessel density was not significantly different between the groups (WT 231.7±3.6 vessels/µm^2^; Flk-1 OE 252.5±8.3 vessels/µm^2^) (Fig. 6D). Thus, blood vessels did not seem to be altered in Flk-1 OE mice hippocampus.

Further, we explored whether increased VEGF signaling in Flk-1 OE mice could have led to gliogenesis [39]. To assess the number of glial cells, we performed a GFAP staining followed by quantification of GFAP-labeled cells in the hilus (Fig. 6E–G). The number of GFAP positive glial cells in the hilus of WT and Flk-1 OE mice was not significantly different (WT 91.8±5.0 cells per hilus; Flk-1 OE 77.4±7.1 cells per hilus) (Fig. 6G). Taken together, our data suggest unaltered glial number in the hilus in Flk-1 OE mice.

## Discussion

Here, using a transgenic mouse line that overexpresses Flk-1 under the Thy-1 promoter, we show that increased levels of Flk-1 in the hippocampus suppress focal seizure susceptibility. In transgenic mice the threshold to generate ADs was significantly increased (twice as high) and the duration of ADs was markedly shortened (less than half) compared to WT animals.

Epileptogenesis, however, was not altered by the overexpression of Flk-1. The Flk-1 OE mice and WT controls did not differ in the number of stimulations needed to reach different seizure stages or the fully kindled state (three stage 5 seizures). Taken together, these results indicate that overexpression of Flk-1 affects focal hippocampal seizure activity without having influence on kindling epileptogenesis or generalized seizure activity (i.e. seizures that have spread outside the focus, here being the hippocampus).

The Flk-1 OE mice exhibited increased expression VEGF, an endogenous ligand of Flk-1. The underlying molecular and cellular mechanism of such increase is currently unclear. A major limitation of traditional transgenic overexpression is the inability to control developmental compensatory or other alterations in signaling pathways. It would be highly warranted to address the question whether Flk-1 overexpression in adult stage (e.g. conditional overexpression, or viral vector-based approach) would also lead to up-regulation of VEGF. In any case, our data suggest that the observed effect on seizures is mediated by an overall increase of VEGF signaling through up-regulation of both Flk-1 and VEGF. Our immunoreactivity analysis indicates mostly neuronal expression of both Flk-1 and VEGF, though possibly in a variety of neuronal populations (see Figs. 4 and 5) In transgenic mice Flk-1 is overexpressed under the Thy-1 promoter, which has been shown to drive transgene expression (Flk-1) selectively in neurons. The identity of the Flk-1 positive and NeuN negative cells is not clear, but may represent a small population of non-neuronal cells due to some unspecificity of the used promoter. The cellular localization of up-regulated VEGF and Flk-1, and how it may influence its seizure modulating effects, remains to be studied in more details.

Our data are in line with previous observations showing that VEGF is able to suppress bicuculline-induced epileptiform discharges in slices from chronic epileptic rats [21], although it was largely unclear which VEGF receptor was responsible for these effects. Our data suggest that Flk-1 might play a major role in the seizure-suppressant effects of VEGF. As we observed a 1.44 fold increase in the expression of VEGF in Flk-1 OE mice, the anti-epileptic effects could be mediated by increased activation of conventional VEGF/Flk-1 signaling pathways described earlier. However, one cannot exclude that the seizure suppressant effect is partly mediated by other VEGF receptors, such as VEGFR-1 (Fms-related tyrosine kinase 1, Flt-1) or neuropilin [40]. It has been shown that VEGF can suppress glutamatergic synaptic transmission in all major synapses in the hippocampus [21], [22]. It is likely that also Flk-1 overexpression exerts similar effect on glutamatergic transmission in the hippocampus, although it needs to be explored.

Recently, a novel concept has been put forward, suggesting that simultaneous overexpression of both the ligand and the receptor of endogenous molecules may exert synergistic seizure-suppressant effect. This concept has been validated for endogenous seizure-suppressant molecules, such as e.g. neuropeptides and their receptors [41], [42]. The finding that in Flk-1 OE mice also VEGF seems to be up-regulated, and potentially strengthens effects of Flk-1 OE, is in line with this conceptual framework.

Another potentially seizure-modulating aspect of Flk-1 overexpression could be the formation of new blood vessels (angiogenesis). It is well known that elevated VEGF signaling may lead to increased levels of vascularization in the brain [35], [36], [43], and increased angiogenesis and vascularization are associated with altered BBB function, neuronal excitability and seizure susceptibility [44], [45], [46]. Therefore, altered vascularization of the hippocampus in Flk-1 OE mice may have contributed to the observed changes in threshold and duration of ADs. However, we were not able to detect any significant alterations in vascularization of the hippocampus in Flk-1 OE mice, possibly due to some compensatory mechanisms. Similarly, no effect of Flk-1 OE has been shown on astrocytes as measured by GFAP immunolabeling, indicating that gliosis did not contribute to seizure-suppressant effects of Flk-1 overexpression. The most likely mechanism of action is modulation of synaptic transmission and plasticity by VEGF, as observed with acute VEGF application in hippocampal slice preparations [21]. However, this hypothesis needs to be tested in future studies.

When considering the seizure modulating effects of VEGF signaling one has to keep in mind that excessive VEGF signaling may exert opposing effects: it could be protective after seizures, but also detrimental [16]. VEGF is for example capable of initiating inflammatory cascades in the brain [39], and could also cause BBB breakdown [47]. Interleukin-1 (IL-1) and tumor necrosis factor-alpha (TNF-α) are up-regulated after seizures, leading to subsequent up-regulation of VEGF [48], [49]. On the other hand, VEGF, while triggering inflammatory cascades, has ability to protect brain cells from various insults. Selectively targeting overexpression of Flk-1 in neurons may avoid unwanted detrimental effects of increased VEGF signaling. Lastly, conventional overexpressing transgenic animals may have some undetected developmental alterations, which could be responsible for the effects observed here. Taken together, these considerations illustrate very complex role of VEGF in cellular and molecular events of normal and pathological conditions, and highlights the need for better understanding of the mechanisms involved in VEGF and its Flk-1 receptor signaling, which may help in developing novel therapeutic strategies for various diseases of the brain, including epilepsy.

### Conclusion

In conclusion, the present study adds new insights on regulation of seizure activity by Flk-1. We have demonstrated that in Flk-1 OE mice the resistance to seizure induction is significantly increased and the duration of epileptic EEG activity decreased. Therefore, targeting Flk-1 receptors may represent a novel approach for optimizing regulation of VEGF signaling pathways in order to modulate excitability in the brain and counteract pathological activity such as in epileptic seizures.
